# Safety and Effectiveness of Inhaling Different Dosage Recombinant Human Interferon *α*1B for Bronchiolitis in Children: a Systematic Review and Meta-Analysis

**DOI:** 10.1155/2022/2229735

**Published:** 2022-04-27

**Authors:** Jiefeng Luo, Mengting Yang, Linan Zeng, Xiangcheng Pan, Dan Liu, Sha Diao, Liang Huang, Ting Chen, Zhi-Jun Jia, Guo Cheng, Qin Yu, Lingli Zhang

**Affiliations:** ^1^West China School of Pharmacy, Sichuan University, Chengdu, China; ^2^Department of Pharmacy, West China Second University Hospital, Sichuan University, Chengdu, China; ^3^Evidence-Based Pharmacy Center, West China Second University Hospital, Sichuan University, Chengdu, China; ^4^Key Laboratory of Birth Defects and Related Diseases of Women and Children, Sichuan University, Ministry of Education, Chengdu, China; ^5^West China School of Medicine, Sichuan University, Chengdu, China; ^6^Department of Pediatrics, West China Second Hospital, Sichuan University, Chengdu, China; ^7^Laboratory of Molecular Translational Medicine, Center for Translational Medicine, Sichuan University, Chengdu, China; ^8^National Drug Clinical Trial Institute, West China Second University Hospital, Sichuan University, Chengdu, China

## Abstract

**Purpose:**

To systematically evaluate the safety and effectiveness of different dosages of recombinant human interferon *α*1b (IFN*α*1b) inhaled for bronchiolitis in children.

**Methods:**

7 databases, including PubMed, EMBASE, Cochrane Library, Web of Science, CNKI, Wanfang Database, and VIP, were searched. The search time was from their inception dates to March 28, 2022. A randomized controlled trial (RCT) of 2 *μ*g/kg IFN*α*1b (low dosage group) monotherapy or in combination with other drugs vs. 4 *μ*g/kg IFN*α*1b (high dosage group) monotherapy or in combination with the other drugs was included. The risk of bias 2.0 evaluated the RCT's quality, and the grading of recommendations assessment, development and evaluation (GRADE) tool was used for evaluating the overall quality of the evidence. Then, a meta-analysis was performed by RevMan 5.4.

**Results:**

A total of 13 RCTs with 1719 children were included. The meta-analysis results showed that the high dosage group was significantly shorter than the low dosage group of the duration of hospital stays (MD = −0.40, 95%CI (−0.73, −0.07), *P* = 0.02) (low quality), three depressions sign disappearing time (MD = −0.60, 95%CI (−1.05, −0.14), *P* = 0.010) (low quality), and wheeze disappearing time (MD = −0.62, 95%CI (−1.17, −0.06),  = 0.03) (low quality). There was no significant difference between the two groups in coughing disappearing time, pulmonary rales disappearing time, wheezing sound disappearing time, or adverse event rates.

**Conclusions:**

Compared with low dosage IFN*α*1b, high dosage IFN*α*1b reduces the duration of hospital stays, the disappearance time of the three depression signs, and the disappearance time of wheeze in the treatment of bronchiolitis in children. Limited by the low quality of the evidence, the conclusions still need to be supported by high-quality studies.

## 1. Introduction

Bronchiolitis is a lower respiratory tract disease that mainly occurs in children under 24 months. The peak age of onset ranges from 2 months to 6 months. The incidence of infants in the first year after birth is approximately 11%, and bronchiolitis is one of the leading causes of illness and hospitalization in children under one year old [1, 2]. The most common etiology of bronchiolitis is respiratory syncytial virus (RSV) infection. Other viruses include the parainfluenza virus, influenza virus, and rhinovirus [2, 3]. Unfortunately, a causative treatment of acute viral bronchiolitis does not exist due to its special pathophysiology [4, 5]. In clinical practice, oxygen and fluid supplementation are usually used for symptomatic treatment [6].

Interferon (IFN) plays an essential role during bronchiolitis, particularly for children infected with RSV [7]. IFNs are a group of signaling proteins synthesized and released by host cells in response to pathogens. Normally, virus-infected cells release IFNs to enable surrounding cells to improve their anti-viral defenses [8]. This early response can influence the clinical course of RSV bronchiolitis, thereby affecting the duration of the disease and damage to the lungs [9]. However, it has been considered that common respiratory viruses, including RSV, may disrupt the host antiviral IFN response [9–12]. Based on the importance of IFN in bronchiolitis, many researchers in China have used exogenous IFN as a supplementary treatment for bronchiolitis.

Recombinant human interferon *α*1B (IFN*α*1B), as a major antiviral subtype in the Chinese population, has attracted the attention of Chinese researchers [5]. As the lesion site of bronchiolitis is located in the bronchioles, administration by nebulized has the advantages of rapid-onset, fewer adverse reactions, and high compliance [5]. Therefore, aerosol inhalation IFN*α*1B for bronchiolitis in children has been recommended by the Chinese National Formulary [13] and the Standardized Management Guidelines for Children's Nebulization Center [14]. At present, the guidelines or expert consensus recommended dosage of nebulized IFN*α*1b for bronchiolitis in children is 2–4 *μ*g/kg [15, 16]. However, there is still controversy about 2 *μ*g/kg or 4 *μ*g/kg in clinical practice. Some researchers believe that 4 *μ*g/kg of IFN*α*1b has better efficacy [17, 18]. Others believe that 2 *μ*g/kg and 4 *μ*g/kg of IFN*α*1b have equivalent efficacy. Considering the safety and economic effects, 2 *μ*g/kg IFN*α*1b should be promoted [19, 20].

Therefore, this study aims to evaluate the safety and effectiveness of inhaling 2 *μ*g/kg and 4 *μ*g/kg of IFN*α*1b for bronchiolitis in children, providing evidence-based evidence for clinical practice.

## 2. Methods

This review was conducted in accordance with the Preferred Reporting Items for Systematic Reviews and Meta-Analyses (PRISMA) guidelines [21].

### 2.1. Search Strategy

A total of 7 databases, including PubMed, Embase (Ovid), Cochrane Library (Ovid), Web of Science, CNKI, Wanfang Database, and VIP, were searched. Additionally, we used a manual search strategy to retrieve the relevant articles cited by the retrieved publications. The search time was from their inception dates to March 28, 2022. Medical subject headings combined with free text terms were used to search for eligible articles. Two clinical trial registration sites, including clinicaltrials.gov (https://clinicaltrials.gov/) and the WHO International Clinical Trials Registry Platform (ICTRP) (http://apps.who.int/trialsearch/), were searched for unpublished but eligible articles.

### 2.2. Inclusion and Exclusion Criteria

#### 2.2.1. The Inclusion Criteria

**Population**: infants under 2 years old and hospitalized with a diagnosis of bronchiolitis.

**Intervention/control**: the high dosage group was given 4 *μ*g/kg IFN*α*1b, and the low dosage group was given 2 *μ*g/kg IFN*α*1b, and both were treated with nebulization. IFN*α*1b monotherapy or in combination with other drugs (e.g., budesonide, albuterol, and hypertonic saline) were included.

**Outcomes**: the primary outcome is the duration of hospital stays. The secondary outcome is based on the main clinical symptoms described in the “Expert consensus on the diagnosis, treatment and prevention of bronchiolitis (2014),” including cough disappearance time, pulmonary rales disappearance time, wheeze disappearance time, and wheezing sound disappearance time, three depression sign disappearance times, and adverse event rates.

#### 2.2.2. The Exclusion Criteria

1. Non-Chinese and Non-English studies
2. Inaccessible studies
3. No information about the child
4. Duplicate publication

### 2.3. Data Extraction and Risk-of-Bias Assessments

Two researchers selected the included RCTs back-to-back according to the inclusion and exclusion criteria and extracted the data. Cochrane risk of bias assessment tool (ROB 2.0) was used to evaluate the RCT's quality, and the grading of recommendations assessment, development and evaluation (GRADE) tool was used to evaluate the overall quality of evidence. When two researchers had opposite opinions, disputes were decided by the third researcher. The content of the literature extraction includes basic information about the literature (such as first author and year of publication); basic information about the child (such as age and course of the disease); basic information about intervention (such as course of treatment, frequency of administration, and combined therapy); and research results (data of outcomes).

### 2.4. Statistical Analysis

RevMan 5.4 statistical software provided by the Cochrane Collaboration Network was performed for meta-analysis. Relative risk (RR) and risk difference (RD) were used for dichotomous data, mean difference (MD) was used for continuous data, and 95% CIs were calculated using the Mantel–Haenszel method and the inverse variance statistical method, respectively. Heterogeneity was tested by *χ*^2^ test and *I*^2^ statistics, and the random effect model was performed for meta-analysis. Subgroup analysis was performed by monotherapy or combined therapy. Sensitivity analysis was performed by eliminating included RCTs one by one in each outcome.

## 3. Results

**Figure 1:**
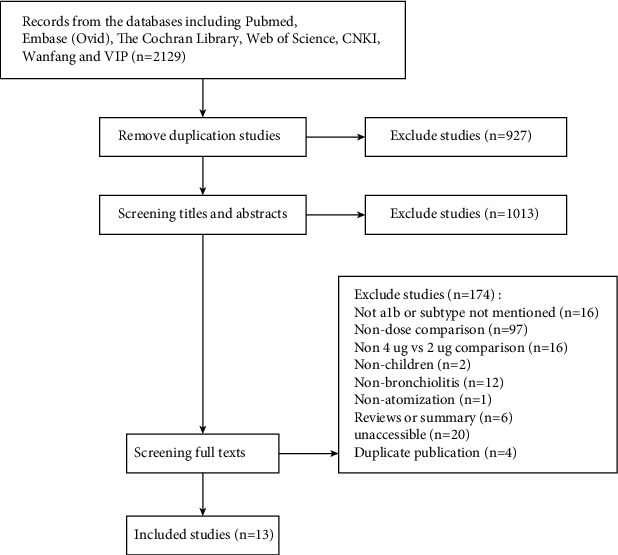
Literature screening flow diagram.

The initial searches included 2129 RCTs. After deduplication, 927 RCTs were removed. After screening titles and abstracts, 1013 RCTs were removed. Ultimately, 13 RCTs remained after screening full texts [17–20, 22–30] (Figure 1).

### 3.1. Characteristics of the Included Studies

**Figure 2:**
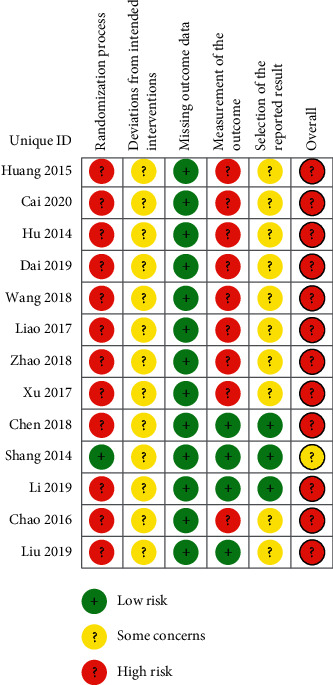
Risk of bias summary.

**Figure 3:**
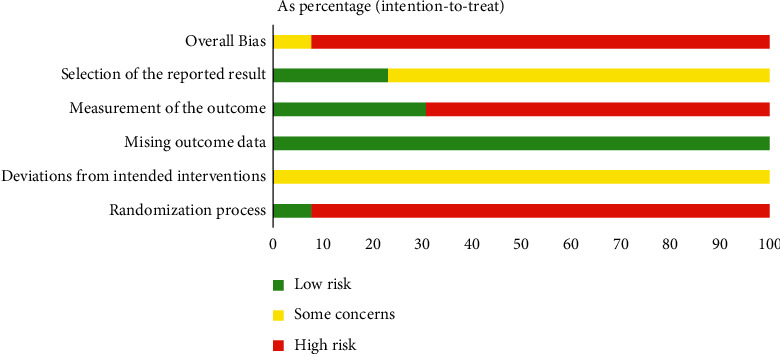
Risk of bias graph.

The basic information of the included RCT is presented in Table 1. Among the included RCTs, 5 RCTs adopted a random number table for randomization [17, 24, 26–28], 1 RCT adopted the envelope method for randomization [18], and the rest did not describe the method or used the wrong method. 1 RCT adopted a central randomization system for concealed allocation [17], and the rest did not mention the method for concealed allocation. Only 1 RCT risk-of-bias assessment result was “Some concerns” [17], and the results of the other RCTs were “High risk”(Figures 2 and 3); GRADE results were “low” and “very low” (Table 2).

Chen 2018 [18] subdivided the drug administration into the early high dosage group, the early low dosage group, the late high dosage group, and the late low dosage group. Since there were no overlap children between early and late, the early and late were independently analyzed. Additionally, Zhao 2018 [17] subdivided the low dosage group into the qid group and the tid group. Since there were no overlap children between the qid group and the tid group, the qid group and the tid group were combined into the low dosage group.

### 3.2. Primary Outcome

**Figure 4:**
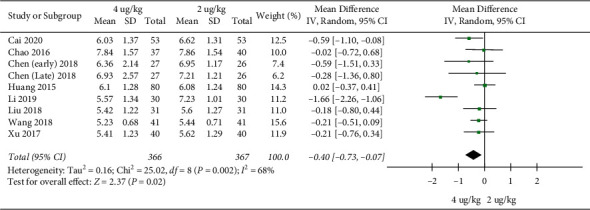
The duration of hospital stays.

**The duration of hospital stays:** 8 RCTs included 733 children [18–20, 22, 23, 26, 27, 29]. The duration of hospital stays in the high dosage group was significantly shorter than that in the low dosage group (MD = −0.40, 95%CI (−0.73,−0.07), *P* = 0.02; *I*^*2*^ = 68%) (low quality) (Figure 4).

### 3.3. Secondary Outcomes

**Figure 5:**
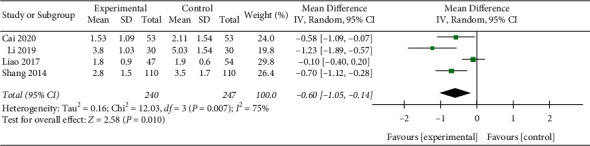
Three depression signs disappearing time.

Three depression signs disappearing time: 4 RCTs included 487 children [17, 18, 22, 28]. Three depressions sign disappearing time in the high dosage group was significantly shorter than that in the low dosage group (MD = −0.60, 95%CI (−1.05,−0.14), *P* = 0.010; *I*^*2*^ = 75%) (low quality) (Figure 5).

**Figure 6:**
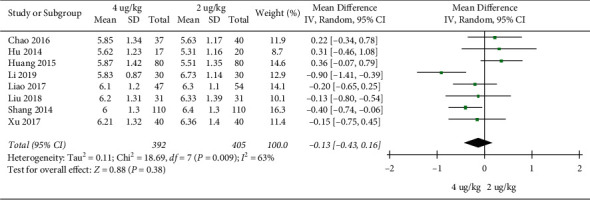
Coughing disappearing time.

Cough disappearance time: 8 RCTs included 797 children [17–19, 23, 27–30]. There was no significant difference in coughing disappearance time between the high dosage group and the low dosage group (MD = −0.13, 95%CI (−0.43, 0.16), *P* = 0.38; I2 = 63%) (very low quality) (Figure 6).

**Figure 7:**
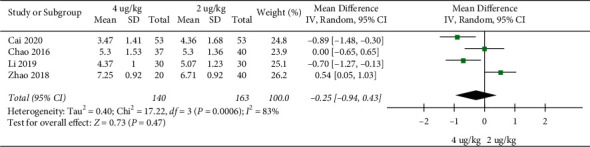
Pulmonary rales disappearing time.

Pulmonary rales disappearing time: 4 RCTs included 303 children [18, 22, 25, 29]. There was no significant difference in pulmonary rales disappearance time between the high dosage group and the low dosage group (MD = −0.25, 95%CI (−0.94, 0.43), *P* = 0.47; I2 = 83%) (very low quality) (Figure 7).

**Figure 8:**
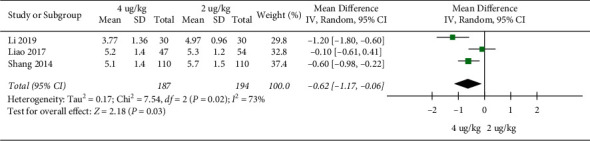
Wheeze disappearing time.

Wheeze disappearing time: 3 RCTs included 381 children [17, 18, 28]. Wheeze disappearing time in the high dosage group was significantly shorter than that in the low dosage group (MD = −0.62, 95%CI (−1.17,−0.06), *P* = 0.03; *I*^*2*^ = 73%) (low quality) (Figure 8).

**Figure 9:**
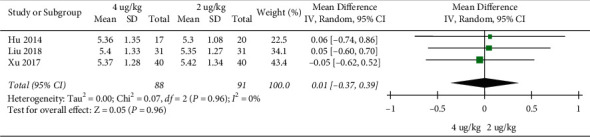
Wheezing sound disappearing time.

Wheezing sound disappearing time: 3 RCTs included 179 children. The high dosage group and the low dosage group had no significant difference in wheezing sound disappearance time (MD = 0.01, 95%CI (−0.37, 0.39), *P* = 0.96; I2 = 0%) (low quality) (Figure 9).

**Figure 10:**
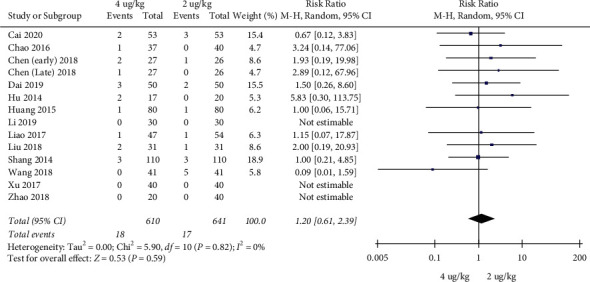
Adverse event rates (RRs).

**Figure 11:**
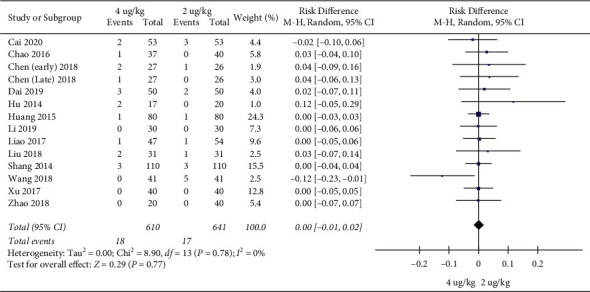
Adverse event rates (RDs).

Adverse event rates: 13 RCTs included 1251 children [17–20, 22–30]. The high dosage group and the low dosage group had no significant difference in adverse event rates (RR = 1.20, 95%CI (0.61, 2.39), *P* = 0.59; RD = 0.00, 95%CI (−0.01, 0.02), *P* = 0.77; I2 = 0%) (low quality) (Figures 10 and 11).

### 3.4. Subgroup Analysis

#### 3.4.1. The Duration of Hospital Stays

**Figure 12:**
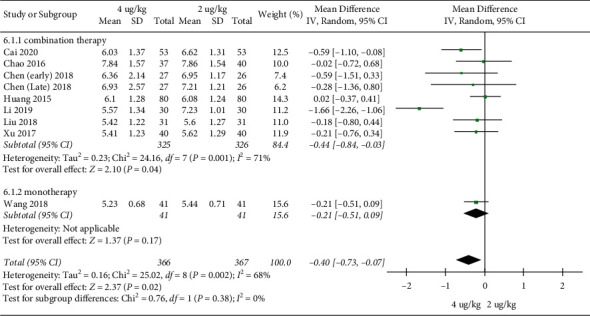
Subgroup analysis (the duration of hospital stays).

The results of subgroup analysis showed that when IFN*α*1b was combined with other drugs, the hospital stay in the high dosage group was significantly shorter than that in the low dosage group (MD = −0.44, 95%CI (−0.84, −0.03), *P* = 0.04; *I*^*2*^ = 71%); when IFN*α*1b was treated with monotherapy, there was no statistically significant difference in hospital stay between the two groups (MD = −0.21, 95%CI (−0.51, 0.09), *P* = 0.17) (Figure 12).

#### 3.4.2. Adverse Event Rates

**Figure 13:**
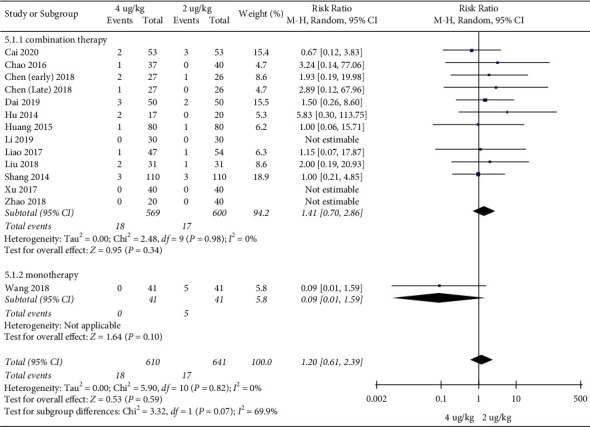
Subgroup analysis (adverse event rates (RRs)).

**Figure 14:**
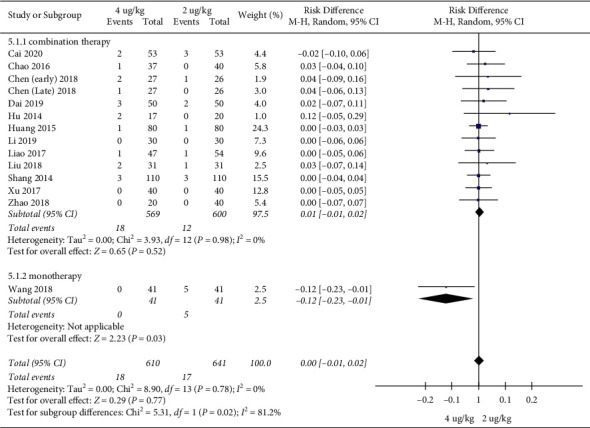
Subgroup analysis (adverse event rates (RDs)).

The results of subgroup analysis showed that regardless of IFN*α*1b combined therapy or monotherapy, there was no statistically significant difference in adverse event rates between the two groups (Figures 13 and 14).

### 3.5. Sensitivity Analysis

When eliminating Li 2019, the heterogeneity of the duration of hospital stays, cough disappearance time, and wheeze disappearance time decreased significantly. The meta-analysis results of the duration of hospital stays, cough disappearance time, three depression sign disappearance times, wheezing sound disappearance times, and adverse event rates showed no change after eliminating including RCTs one by one. The meta-analysis results of pulmonary rales disappearing time turned into statistically significant differences (MD = −0.55, 95%CI (−1.06,−0.04)) when eliminating Zhao 2018. The meta-analysis results of wheeze disappearing time turned into no statistically significant difference (MD = −0.38, 95%CI (−0.87, 0.11)) when eliminating Li 2019.

## 4. Discussions

### 4.1. Summary

Bronchiolitis is a lower respiratory tract infection mainly involving small airways (bronchioles). It is a common cause of illness and hospitalization in infants and young children. Severe bronchiolitis may also increase the risk of children developing asthma and continue to adulthood [6, 31, 32]. Therefore, the etiological treatment of bronchiolitis is of great importance.

This study included 13 RCTs with 1719 children to study the safety and effectiveness of different dosages of IFN*α*1b inhalation for bronchiolitis. The results showed that in the duration of hospital stays, wheeze disappearing time, and three depressions sign disappearing time, the high-dosage group is significantly shorter than the low-dosage group (*P* ≤ 0.05), but there was no difference between the two groups in cough disappearance time, pulmonary rales disappearing time, wheezing sound disappearing time, and adverse event rates (*P* > 0.05).

### 4.2. Comparison with Similar Research

Chen Can [33] conducted a meta-analysis on the effectiveness and safety of IFN*α*1b nebulized inhalation for bronchiolitis based on 24 RCTs. The study results concluded that IFN*α*1b nebulized inhalation is safe and effective for bronchiolitis. However, there are still some problems in this study. The first is the high heterogeneity, such as the outcome of the duration of hospital stays (*P*＜0.01, *I*^*2*^ = 98%), and the subgroup analysis of different doses of IFN*α*1b did not reduce the heterogeneity. In addition, only 3 RCTs compared different dosages of IFN*α*1b [17, 19, 30], and different dosages of IFN*α*1b were not further studied.

### 4.3. Sensitivity Analysis

When eliminating Li 2019 [18], the heterogeneity of each outcome has decreased, and the meta-analysis results of wheeze disappearance time have changed. The possible reason is that the disease course of children in this RCT (both groups are ＜24 hours) is significantly shorter than in other RCTs (1–6 days). Another cause may be that the children have a high proportion of fever. The proportion of children with a temperature ≥39°C in the high and low dosage groups is 30% and 43%, respectively. However, the consensus points out that the temperature of children with bronchiolitis generally does not exceed 39°C [3]. When eliminating Zhao (2018), the meta-analysis results of pulmonary rales disappearing time have changed. The possible reason is that Zhao (2018) [25] subdivides the low dosage group into three times a day and two times a day. However, it is unified into the low dosage group for statistical analysis in this study, which may lead to a change in baseline comparability. The difference in sample size between the high dosage group and the low dosage group may also be one of the reasons.

### 4.4. Subgroup Analysis

Subgroup analysis showed no significant difference between the high dosage group and the low dosage group in the duration of hospital stays and adverse event rates when IFN*α*1b was treated with monotherapy. In terms of the duration of hospital stays, the conclusions of monotherapy and when combined with other drugs are inconsistent. However, considering that there is only 1 RCT on monotherapy, it cannot be concluded that there is no statistically significant difference in the duration of hospital stays between the high dosage group and the low dosage group when IFN*α*1b was treated with monotherapy. A large sample, multicenter clinical research is needed to support these results.

### 4.5. Risk-of-Bias Assessments

The areas of lower quality are “Randomization process” and “Randomization process,” 53.85% of the RCTs did not use or used the wrong method for randomization, such as according to the order of admission, which may lead to incomparable baselines between the two groups. In addition, 92.31% of the RCTs did not use blinding or concealed allocation, which may lead to bias when giving interventions or measuring outcome data.

## 5. Advantages and Limitations

### 5.1. Advantage

There has been controversy about the different dosages of IFN*α*1b inhaling for bronchiolitis in clinical practice. However, there is not any evidence-based medical evidence. This study uses systematic reviews and meta-analysis methods to provide a reference for clinical practice.

### 5.2. Limitation

There are some limitations to this study. First, the quality of the included RCTs is low. Only 1 RCT has a result of “Some concerns,” and the others are “High risk.” Second, most of the outcomes of this study adopt the disappearance time of disease symptoms, and the judgment criteria are subjective. Third, since none of the included RCTs mentioned the symptom classification of bronchiolitis, the disease severity was not considered in this study.

## 6. Conclusions

Compared with the low dosage IFN*α*1b, the high dosage IFN*α*1b reduces the duration of hospital stays, the disappearance time of the three depression signs, and the disappearance time of wheeze in the treatment of bronchiolitis in children. Limited by the quality of the included RCTs, the above conclusions still need to be supported by large samples and high-quality studies.

## Figures and Tables

**Table 1:** Basic information of included RCTs.

Study ID	Age (months)	Course of disease (days)	Number		Interventions				Outcome
			High dosage group	Low dosage group	High dosage group	Combination with other drugs	Low dosage group	Combination with other drugs	
Huang 2015 [12]	T: 9.23 ± 0.53 C: 9.45 ± 0.62	T: 6.08 ± 1.24 C: 6.10 ± 1.28	80	80	IFN*α*1b 4 *μ*g/kg qid, 5–7d	Albuterol + budesonide	IFN*α*1b 2 *μ*g/kg, qid, 5–7d	Albuterol + budesonide	①③⑦
Shang 2014 [10]	—	—	110	110	IFN*α*1b 4 *μ*g/kg, qid, 5–7d	Albuterol + budesonide	IFN*α*1b 2 *μ*g/kg, qid, 5–7d	Albuterol + budesonide	②③⑤⑦
Li 2019 [11]	T: 11.83 ± 2.53 C: 11.63 ± 2.17	T: 0.90 ± 0.34 C: 0.92 ± 0.35	30	30	IFN*α*1b 4 *μ*g/kg, qid, 5–7d	Albuterol + budesonide	IFN*α*1b 2 *μ*g/kg, qid, 5–7d	Albuterol + budesonide	①②③④⑤⑦
Cai 2020 [14]	6.54 ± 3.10	—	53	53	IFN*α*1b 4 *μ*g/kg, qid, 5–7d	Albuterol + budesonide	IFN*α*1b 2 *μ*g/kg, qid,5–7d	Albuterol + budesonide	①②④⑦
Hu 2014 [22]	—	—	17	20	IFN*α*1b 4 *μ*g/kg, qid, 5–7d	Albuterol + budesonide	IFN*α*1b 2 *μ*g/kg, qid,5–7d	Albuterol + budesonide	③⑥⑦
Dai 2019 [16]	T: 13.52 ± 3.87 C: 13.63 ± 3.84	T: (5.36 ± 0.74) C: (5.11 ± 0.77)	50	50	IFN*α*1b 4 *μ*g/kg, qid, 7d	Albuterol + budesonide	IFN*α*1b 2 *μ*g/kg, qid,7d	Albuterol + budesonide	⑦
Wang 2018 [13]	T: 10.42 ± 0.56 C: 10.31 ± 0.77	T: (2.46 ± 0.68) C: (2.29 ± 0.77)	41	41	IFN*α*1b 4 *μ*g/kg, qid, 5–7d	—	IFN*α*1b 2 *μ*g/kg, qid, 5–7d	—	①⑦
Liao 2017 [20]	T: 9.4 ± 0.5 C: 9.6 ± 0.6	—	47	54	IFN*α*1b 4 *μ*g/kg, qid	Budesonide + ipratropium bromide	IFN*α*1b 2 *μ*g/kg, qid	Budesonide + ipratropium bromide	②③⑤⑦
Zhao 2018 [17]	T1: 6.69 ± 0.6 T2: 7.44 ± 0.96 C: 7.2 ± 0.84	—	20	40	IFN*α*1b 4 *μ*g/kg, qid, 5–7d	Albuterol + budesonide	IFN*α*1b 2 *μ*g/kg, qid, 5–7d	Albuterol + budesonide	④⑦
Xu 2017 [19]	T: 12.21 ± 3.32 C: 13.23 ± 3.45	T: 2.57 ± 0.42 C: 2.51 ± 0.52	40	40	IFN*α*1b 4 *μ*g/kg, qid, 7d	Budesonide + ambroxol	IFN*α*1b 2 *μ*g/kg, qid, 7d	Budesonide + ambroxol	①③⑥⑦
Liu 2018 [15]	T: 17.16 ± 6.12 C: 18 ± 2.76	T: 2.57 ± 0.42 C: 2.51 ± 0.52	31	31	IFN*α*1b 4 *μ*g/kg, qid, 7d	Budesonide + ambroxol	IFN*α*1b 2 *μ*g/kg, qid, 7d	Budesonide + ambroxol	①③⑥⑦
Chao 2016 [21]	—	1–7	37	40 (tid 20; qid 20)	IFN*α*1b 4 *μ*g/kg, qid, 5–7d	Budesonide + terbutaline + normal saline	IFN*α*1b 2 *μ*g/kg, qid, 5–7d	Budesonide + terbutaline + normal saline	①③④⑦
Chen 2018 [18]	T (early):6.0 ± 1.8 C (early):5.5 ± 2.1 T (late):6.9 ± 3.6 C (late):6.8 ± 2.9	—	54 (early 27; late 27)	52 (early 26; late 26)	IFN*α*1b 4 *μ*g/kg, qid, 5–7d	Budesonide + terbutaline	IFN*α*1b 2 *μ*g/kg, qid, 5–7d	Budesonide + terbutaline	①⑦

①Qid: twice a day; tid: three times a day; ①the duration of hospital stays; ② three depression sign disappearing times; ③ cough disappearance time; ④ pulmonary rales disappearing time; ⑤ wheeze disappearing time; ⑥ wheezing sound disappearing time; ⑦ adverse event rates. T: high dosage group; C: low dosage group.

**Table 2:** GRADE evidence profile.

Certainty assessment							No of patients		Effect		Certainty
Outcome	Study design	Risk of bias	Inconsistency	Indirectness	Imprecision	Other considerations	2ug/kg IFN*α*1b	4ug/kg IFN*α*1b	Relative (95% CI)	Absolute (95% CI)	
①	RCT	Very serious a	Not serious	Not serious	Not serious	None	366	367	—	MD 0.40 lower (0.73 lower to 0.07 lower)	⊕⊕○○ LOW
②	RCT	Very serious a	Not serious	Not serious	Not serious	None	240	247	—	MD 0.60 lower (1.05 lower to 0.14 lower)	⊕⊕○○ LOW
③	RCT	Very serious a	Serious b	Not serious	Not serious	None	392	405	—	MD 0.13 lower (0.43 lower to 0.16 higher)	⊕○○○ VERY LOW
④	RCT	Very serious a	Serious b	Not serious	Not serious	None	140	163	—	MD 0.25 lower (0.94 lower to 0.43 higher)	⊕○○○ VERY LOW
⑤	RCT	Very serious a	Not serious	Not serious	Not serious	None	187	194	—	MD 0.62 lower (1.17 lower to 0.06 lower)	⊕⊕○○ LOW
⑥	RCT	Very serious a	Not serious	Not serious	Not serious	None	88	91	—	MD 0.01 higher (0.37 lower to 0.39 higher)	⊕⊕○○ LOW
⑦	RCT	Very serious a	Not serious	Not serious	Not serious	None	18/610 (3.0%)	17/641 (2.7%)	RR 1.07 (0.58 TO 1 .98)	2 more per 1,000 (from 11 fewer to 26 more)	⊕⊕○○ LOW

**CI:** confidence interval; **SMD:** standardized mean difference; **RR:** risk ratio; **RD**: risk difference. a. Using a wrong method for randomization or did not perform allocation conceal; b. Included RCTs were distributed on both side of the invalidity line. ① The duration of hospital stays; ② three depressions sign disappearing time; ③ cough disappearance time; ④ pulmonary rales disappearing time; ⑤ wheeze disappearing time; ⑥ wheezing sound disappearing time; ⑦ adverse event rates.

## Data Availability

The RevMan data used to support the findings of this study are available from the corresponding author upon request.

## Supplementary Materials

Supplementary Materials Search strategies for all databases can be seen in the supplementary material.
