# Supplementary material for: Safety and Effectiveness of Inhaling Different Dosage Recombinant Human Interferon α1B for Bronchiolitis in Children: a Systematic Review and Meta-Analysis
Source: Evid Based Complement Alternat Med. 2022 Apr 27;2022:2229735. doi: 10.1155/2022/2229735 (PMC9068289; doi:10.1155/2022/2229735)
Supplement: Supplementary Materials — Search strategies for all databases can be seen in the supplementary material. [file 2229735.f1.docx]

|  | **Pubmed** | **Embase** | **Cochrane Library** | **WOS** |
| --- | --- | --- | --- | --- |
| **Time** | 2022.3.28 | 2022.3.28 | 2022.3.28 | 2022.3.28 |
| **Search strategy** | 1. "bronchiolitis"[MeSH Terms] OR "bronchiolitis"[All Fields] OR "bronchiolitides"[All Fields] OR "respiratory sounds"[MeSH Terms] OR "wheez*" [TW] OR "respiratory syncytial viruses"[MeSH Terms] OR "respiratory syncytial viruses"[All Fields] OR "RSV"[All Fields] OR "paramyxoviridae infections"[MeSH Terms] OR "parainfluenzae"[All Fields] OR "rhinovirus"[MeSH Terms] OR "rhinovirus"[All Fields] OR "adenoviridae"[MeSH Terms] OR "adenoviridae"[All Fields] OR "influenza, human"[MeSH Terms] 2. "interferons"[MeSH Terms] OR "IFN"[All Fields] OR "pegylated interferon"[All Fields] OR " peginterferon"[All Fields] OR "alpha 1b interferon"[All Fields] OR "alpha1 interferon"[All Fields] OR "alpha interferon "[All Fields] 3. "nebuli*"[All Fields] OR "vapor*"[All Fields] OR "vapour*"[All Fields] OR "atomi*"[All Fields] OR "inhal*"[All Fields] OR "aerosol*"[All Fields] 4. 1 AND 2 AND 3 | 1. exp bronchiolitis/  2. exp Respiratory Sounds/  3. exp Respiratory Syncytial Viruses/  4. exp Paramyxoviridae Infections/  5. exp Rhinovirus/  6. exp Adenoviridae/  7. exp Influenza, Human/  8. (bronchiolitis or bronchiolitides or wheez* or respiratory syncytial viruses or RSV or parainfluenzae or rhinovirus or adenoviridae).ti,ab.  9. exp Interferons/  10. (IFN or pegylated interferon or peginterferon or alpha 1b interferon or alpha 1 interferon or alpha interferon).ti,ab.  11. (nebuli* or vapor* or vapour* or atomi* or inhal* or aerosol*).ti,ab.  12. 1 or 2 or 3 or 4 or 5 or 6 or 7 or 8  13. 9 or 10  14. 11 and 12 and 13 | 1. exp bronchiolitis/  2. exp Respiratory Sounds/  3. exp Respiratory Syncytial Viruses/  4. exp Paramyxoviridae Infections/  5. exp Rhinovirus/  6. exp Adenoviridae/  7. exp Influenza, Human/  8. (bronchiolitis or bronchiolitides or wheez* or respiratory syncytial viruses or RSV or parainfluenzae or rhinovirus or adenoviridae).ti,ab.  9. exp Interferons/  10. (IFN or pegylated interferon or peginterferon or alpha 1b interferon or alpha 1 interferon or alpha interferon).ti,ab.  11. (nebuli* or vapor* or vapour* or atomi* or inhal* or aerosol*).ti,ab.  12. 1 or 2 or 3 or 4 or 5 or 6 or 7 or 8  13. 9 or 10  14. 11 and 12 and 13 | TS=(bronchiolit* or wheez* or "respiratory syncytial virus" or "respiratory syncytial viruses" or rsv or parainfluenza* or "respirovirus infection" or "respirovirus infections" or rhinovirus* or adenovirus* or influenza*) AND TS=(“interferon” or “IFN” or “pegylated interferon” or “peginterferon” or “alpha1b interferon” or “alpha1 interferon” or “alpha interferon”) AND TS=(nebuli* or vapor* or vapour* or atomi* or inhal* or aerosol*) |
